# The combined effect of *PDX1,* epidermal growth factor and poly-L-ornithine on human amnion epithelial cells’ differentiation

**DOI:** 10.1186/s12861-016-0108-y

**Published:** 2016-04-12

**Authors:** Shruti Balaji, Yu Zhou, Anasuya Ganguly, Emmanuel C. Opara, Shay Soker

**Affiliations:** Wake Forest Institute for Regenerative Medicine, Winston-Salem, NC 27101 USA; Virginia Tech-Wake Forest University School of Biomedical Engineering & Sciences, Wake Forest School of Medicine, Winston-Salem, NC 27157 USA; Birla Institute of Technology & Science, Pilani K K Birla Goa campus, Zuari Nagar, 403726 Goa India

**Keywords:** Amnion epithelial cells, Pancreas, Differentiation, PDX1, Epidermal Growth Factor, Poly-L-Ornithine

## Abstract

**Background:**

It has been suggested that the ectopic expression of PDX1, a dominant pancreatic transcription factor, plays a critical role in the developmental programming of the pancreas even from cells of unrelated tissues such as keratinocytes and amniotic fluid stem cells. In this study we have chosen to drive pancreatic development in human amnion epithelial cells by inducing endogenous *PDX1* expression. Further, we have investigated the role of Epidermal Growth Factor (EGF) and Poly-L-Ornithine (PLO) on this differentiation process.

**Results:**

Human amnion epithelial cells expressed high levels of endogenous *PDX1* upon transduction with an adenoviral vector expressing murine *Pdx1*. Other markers of various stages of pancreatic differentiation such as *NKX6.1*, *SOX17*, *RFX6*, *FOXA2, CFTR*, *NEUROD1*, *PAX4* and *PPY* were also expressed upon *Pdx1* transduction.

Although initial expression of pancreatic progenitor markers was higher in culture conditions lacking EGF, for a sustained and increased expression EGF was required. Culture on PLO further increased the positive impact of EGF.

**Conclusion:**

Pancreatic marker expression subsequent to m*Pdx1* transduction suggests that this approach may facilitate the in vitro differentiation of hAECs into cells of the endocrine pancreas. This result may have important implications in diabetes therapy.

**Electronic supplementary material:**

The online version of this article (doi:10.1186/s12861-016-0108-y) contains supplementary material, which is available to authorized users.

## Background

The global incidence of diabetes is expected to cross the 300 million mark by 2030 [1]. With current treatment strategies being inadequate in addressing the complications associated with the disease [2], an alternative cell-based therapy approach is urgently needed. The Edmonton protocol was the first to provide a proof-of-concept of a cell-based therapy for diabetes [3]. However, due to a severe shortage of donor pancreases as well as other complications associated with the procedure itself such as the need for life-long immunosuppression and failure to achieve complete insulin independence [4], alternative approaches need to be examined. Multipotent cells from foetal tissue may provide one such alternative.

To date, attempts have been made to differentiate amnion epithelial cells (AECs), placenta-derived multipotent progenitor cells and amniotic fluid stem cells down the pancreatic lineage [5–9]. However, multiple protocols exist for the differentiation of the same cell type. Further, the molecular basis of the differentiation process and individual media components have not yet been studied. The present study aims to bridge this gap in current research.

The pancreatic and duodenal homeobox-1 (Pdx-1) is a master regulator of pancreatic development and differentiation [10, 11]. It is responsible for the differentiation of progenitor cells into cells of the endocrine pancreas. The complex ductal architecture of the pancreas is also established by a reiterative program of branching morphogenesis which proceeds concomitantly with peak Pdx1 transcription factor expression [12]. PDX1 has been suggested to play a critical role in the developmental re-programming of the pancreas from cells of unrelated tissues such as keratinocytes and amniotic fluid stem cells [6, 13]. Furthermore, induced PDX1 expression in human and non-human primate amniotic fluid-derived stem cells (AFSCs) cultured on PLO-coated plates, was shown to initiate pancreatic differentiation [3, 4]. PDX1 is thus, an important factor in the development of both the pancreatic islet and ductal cells.

Epidermal growth factor (EGF) is a standard component of human amnion epithelial cell growth media as it improves the growth of these cells [14, 15]. In the developing pancreas, EGF increases the mass of pancreatic epithelial cells whereas a lack of it promotes endocrine differentiation [16, 17]. When administered to adult pigs, excess of EGF leads to pancreatic duct hyperplasia [18]. One aspect of this study therefore is to study the effect of EGF on hAECs that are being differentiated down the pancreatic lineage.

PLO is a synthetic amino acid chain that is positively charged and widely used as a coating to enhance cell attachment and adhesion to both plastic ware and glass surfaces. PLO has previously been used in the differentiation of embryonic stem cells into insulin-producing clusters [19, 20].

In this study, we have used ectopic expression of m*Pdx1* as a potential approach for the differentiation of hAECs into pancreatic progenitors. We found that endogenous *PDX1* expression was induced several fold upon m*Pdx1* transduction. Several other genes that are expressed by pancreatic progenitor cells such as *SOX17*, *RFX6*, *CFTR* and *FOXA2* were also expressed. The presence of EGF and PLO in the culture environment potentiated this expression. A two-tailed *t*-test was used to determine the statistical significance of the observed changes in gene dynamics.

## Results

**Fig. 1:**
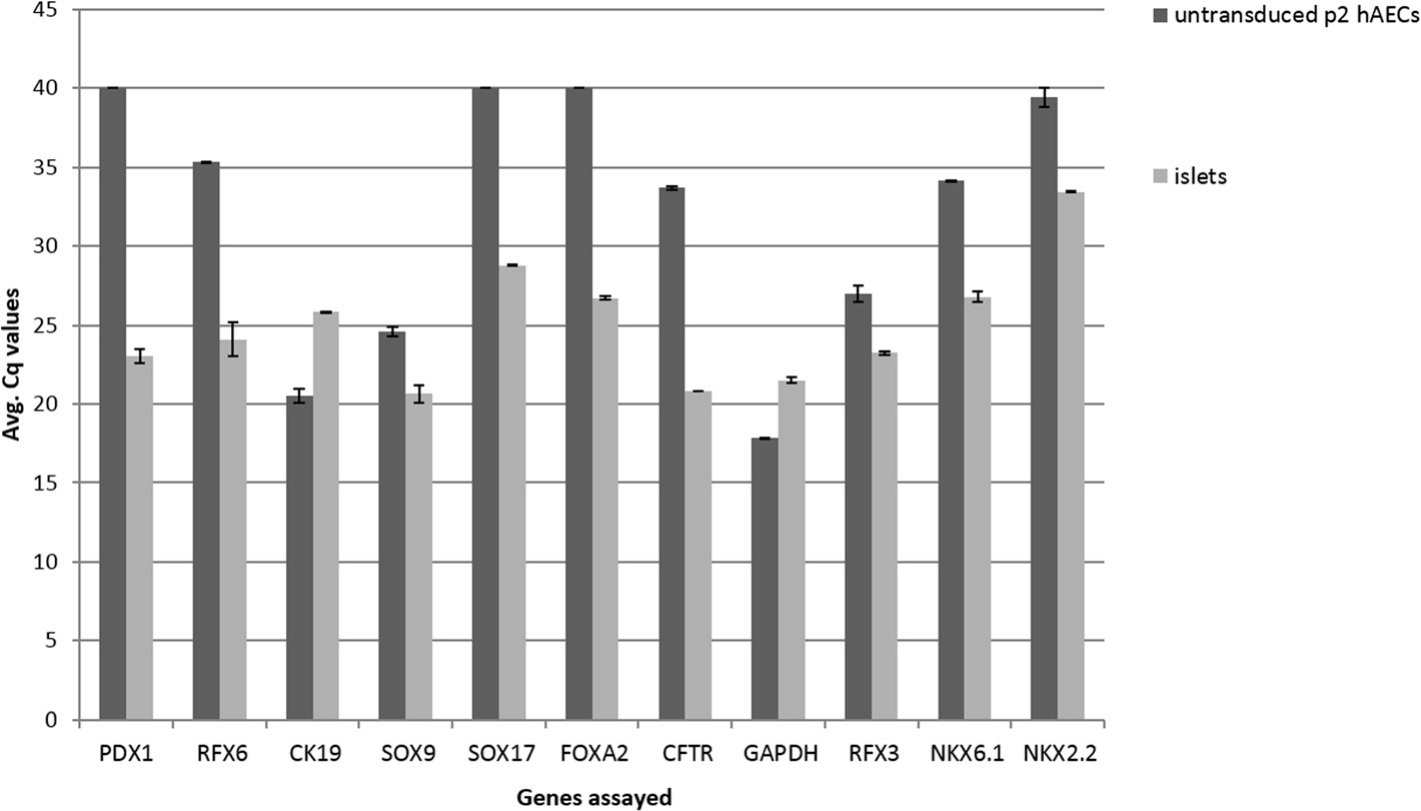
Comparison of average Cq values between untransduced p2 hAECs and crude human islet preparations for pancreatic progenitor-specific genes. Pancreatic progenitor-specific genes were assayed by means of qPCR for untransduced p2 hAECs (baseline gene expression) and crude human islet preparations (positive control). Error bars represent mean ± SD. It can be noted that endogenous *CK19* and *SOX9* expression is high in untransduced hAECs while expression of *PDX1*, *RFX6, SOX17, FOXA2* and *NKX2.2* is virtually absent (Cq > 35—gene expression undetectable, Cq 30–35—low level of gene expression, Cq 25–30—high level of gene expression, Cq <25—very high level of gene expression)

Baseline expression of pancreatic regulatory genes in hAECs (passage 2) showed high *CK19* and *SOX9* expression (Cq <25) (Fig. 1). In fact *CK19* expression was higher in hAECs compared to adult human islets. Moderate *RFX3* expression (Average Cq 27) was also observed. Expression of all other genes that were tested was either very low or absent in hAECs (Cq ≥35). We therefore attempted to initiate the process of pancreatic differentiation of hAECs by transient transduction of m*Pdx1*, a critical factor in the development of the pancreas.

### Effect of m*Pdx1* transduction

Transduction of hAECs with non-integrating, recombinant adenovirus harbouring the mouse *Pdx1* gene was successful. Transduction efficiency increased in a dose- and time-dependent manner from 10 to 200 multiplicity of infection (MOI) of the adenoviruses (Additional file 1). However, since higher concentrations of the adenoviral vector caused increased cell death over an extended culture period, we performed all subsequent experiments with 50 MOI of the virus. Transduction efficiency was 12 % at 24 h and 69 % at 48 h at this viral titre.

**Fig. 2:**
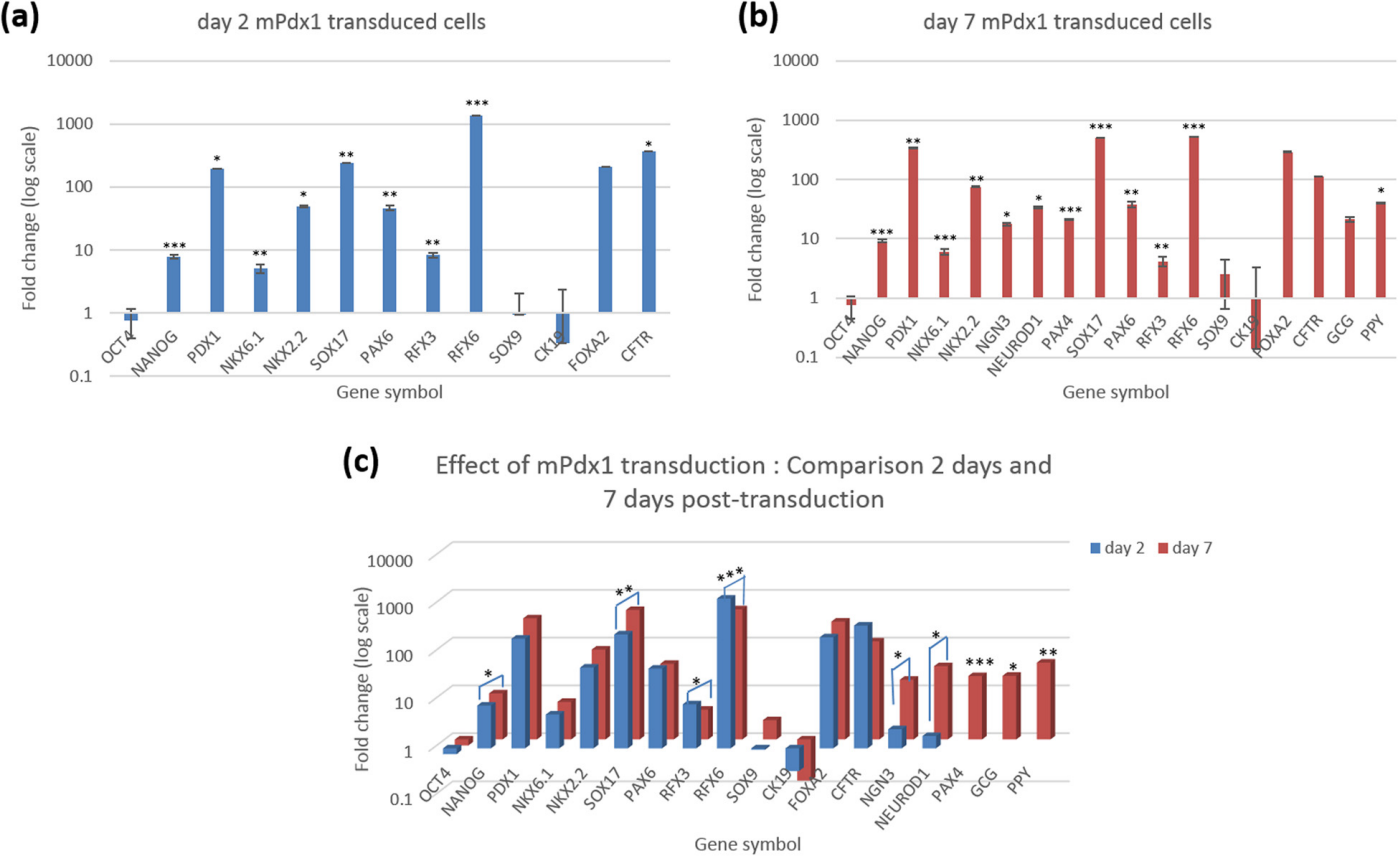
Effect of adenoviral transduction of human amnion epithelial cells on pancreatic marker gene expression. Fold-change expression of human pancreatic marker genes was assayed by means of qPCR (**a**) 2 days and (**b**) 7 days after m*Pdx1* adenoviral transduction of p2 hAECs. Fold-change is relative to untreated cells. Error bars represent mean ± SD. Only those genes with a Cq <35 are represented here. Of the 21 genes assayed, expression of 13 genes on day 2 and 18 genes on day 7 were detected. (**c**) Five genes in particular- *NANOG, SOX17, NGN3, NEUROD1* and *PAX4*—showed significant increase in expression while *RFX6* and *RFX3* showed a significant loss of expression over the 7 day period. (* *p* < 0.05, ** *p* < 0.005, *** *p* < 0.0005; *n* = 4)

Subsequent to m*Pdx1* transduction, there was a dramatic increase in expression of endogenous human *PDX1*. At day 2, expression was almost 200-fold higher than untransduced controls (Fig. 2a). Expression increased marginally up to 7 days post-transduction. The expression of the *PDX1* gene was confirmed by comparing *PDX1* gene expression in cells that were transduced with a control EGFP adenovirus. EGFP transduction did not cause expression of *PDX1* (Data not shown).

Expression of *PDX1* in turn caused many other pancreatic marker genes to be expressed (Additional file 2). In particular, there was a greater than 200-fold increase in *SOX17* expression and a greater than 1000-fold increase in *RFX6* expression in m*Pdx1* transduced cells as compared to untransduced controls on day 2 post-transduction (Fig. 2a). Although there was a further 2-fold increase in *SOX17* expression, *RFX6* expression dropped by approximately 3-fold at the end of the culture period. *RFX3* expression also dropped significantly by the 7th day (Fig. 2c). Interestingly, the pancreatic endocrine progenitor markers- *NGN3*, *NEUROD1* and *PAX4* began to be expressed by day 7, albeit at low levels (Fig. 2b). Significant expression of markers of β-cell committed cells viz. *NKX6.1*, *NKX2.2* and *PAX6* were also observed at the 2 day and 7 day time points although the difference in expression of these genes between the two time points was not significant. Further, expression of adult endocrine pancreas marker genes, *GCG* and *PPY*, also began by day 7 although this seemed to be more of an effect of the adenoviral transduction itself because even EGFP expressing cells expressed these two markers.

Microscopically, it was observed that cell proliferation ceased upon transduction with m*Pdx1*. This was not the case with control cells transduced with EGFP (Additional file 3).

Expression of RFX6 and RFX3 at the protein level was confirmed by Western blot analysis (Additional file 4).

### Effect of EGF on m*Pdx1* transduction

Since previous reports have suggested that an environment lacking EGF drives the pancreatic differentiation process forward, we investigated if this is indeed the case for hAECs transduced with m*Pdx1*.

**Fig. 3:**
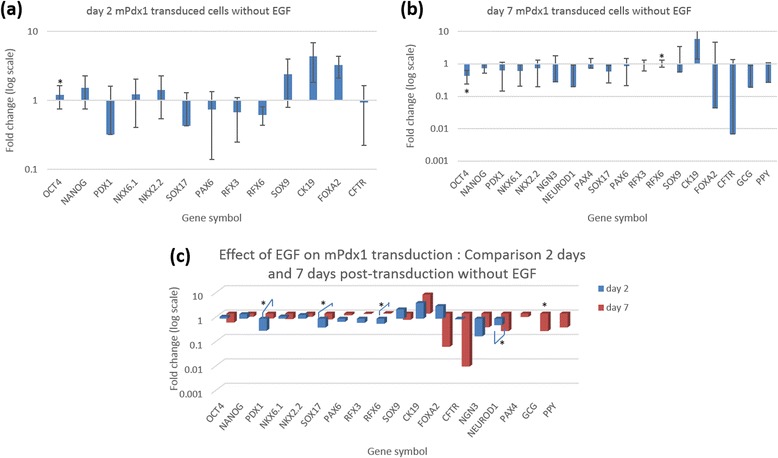
Effect of EGF on m*Pdx1* transduction. The expression of human pancreatic marker genes by m*Pdx1*-transduced hAECs in a culture environment lacking EGF was assayed by means of qPCR over a 7 day period. (**a**) Gene expression in cells grown in the absence of EGF for 2 days (**b**) Gene expression in cells grown in the absence of EGF for 7 days (**c**) Comparison of gene expression in cells grown in the absence of EGF for 2 and 7 days. Fold change was compared to cells grown in the presence of 10 ng/mL EGF. Although it appeared that there was a loss of gene expression across the panel by the 7th day, this loss was not significant when compared to expression in cells grown in an environment containing EGF. (* *p* < 0.05, ** *p* < 0.005, *** *p* < 0.0005; *n* = 2). Error bars represent mean ± SD

In general it was observed that a lack of EGF in the culture medium, did not make any statistically significant difference to gene expression (Fig. 3 and Additional file 5) although by day 7 there seemed to be a loss of gene expression across the marker panel, including the endocrine pancreas markers. However this was observed as a less than 1-fold change in expression in 16 of 18 genes tested. Subsequent experiments therefore continued to be carried out in the presence of 10 ng/mL EGF.

### Combined effect of EGF and PLO on m*Pdx1* transduction

Pancreatic differentiation experiments with AFSCs have previously shown low level of insulin mRNA expression only in cells grown on a PLO coating [5]. We wanted to check therefore if growing the adenovirally transduced hAECs on PLO-coated plates would potentiate the process of pancreatic differentiation.

**Fig. 4:**
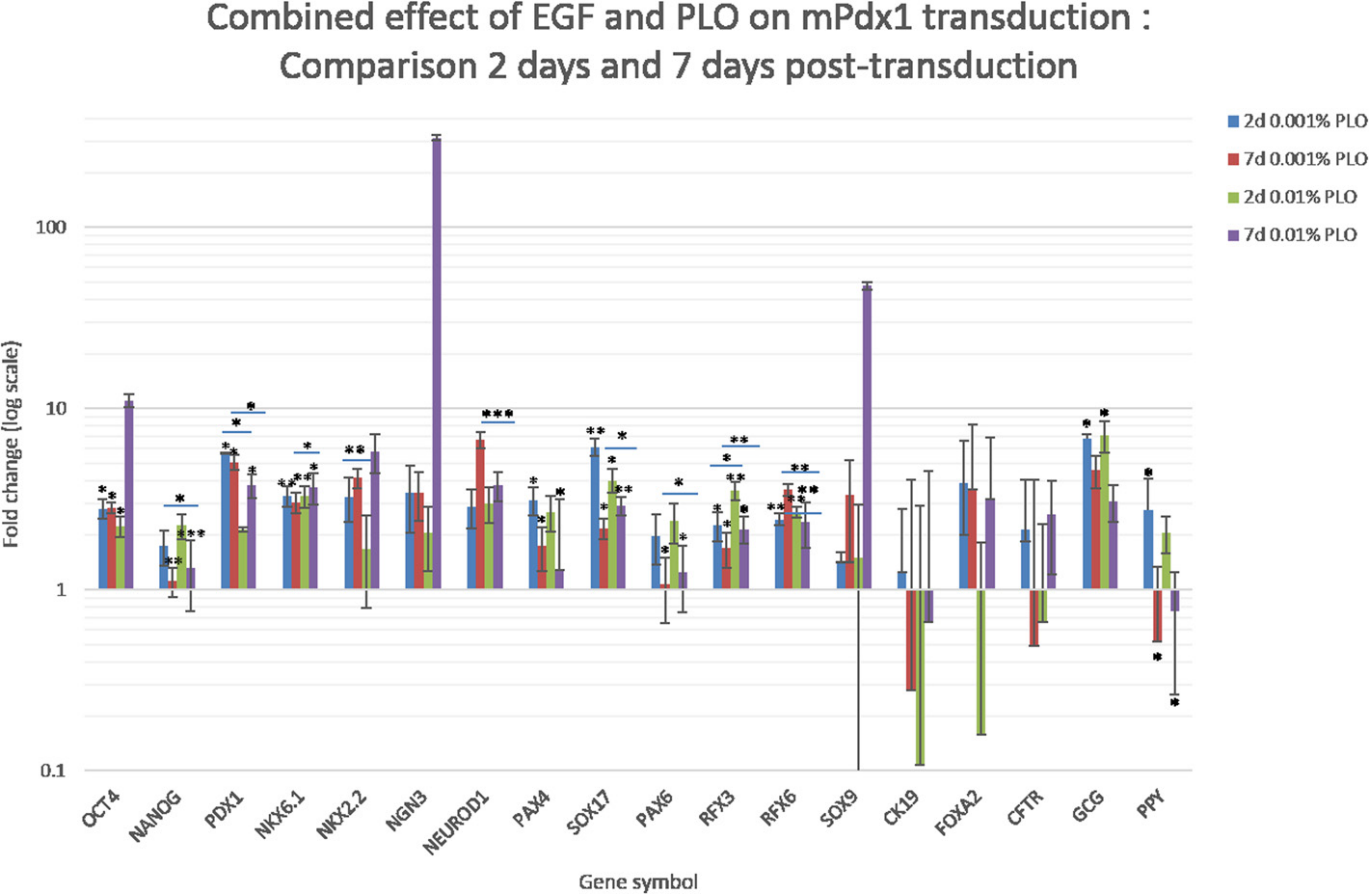
Combined effect of EGF and PLO on m*Pdx1* transduction. Two days and 7 days after m*Pdx1* adenoviral transduction of p2 hAECs grown in the presence of EGF and PLO, the expression of human pancreatic marker genes was assayed by means of qPCR. Expression was compared to cells that were cultured only in the presence of EGF. mRNA expressions were normalized to endogenous *GAPDH* expression. Similar to transduced cells cultured with EGF alone, the 7 genes- *NKX2.2, PAX6, PDX1, RFX6*, *SOX17, CFTR* and *FOXA2*—showed a dramatic and higher change in expression upon m*Pdx1* transduction. In most cases, expression of genes in cells grown on PLO was higher than their non-PLO counterpart. (* *p* < 0.05, ** *p* < 0.005, *** *p* < 0.0005; *n* = 2)

It was observed that the gene expression dynamics of cells growing on PLO was more or less the same irrespective of the concentration. However culture on 0.001 % PLO resulted in a statistically significant higher expression of most genes (Additional file 6). Specifically, expression of endogenous *PDX1* was higher in cells cultured on 0.001 % PLO on both days as compared to their non-PLO counterparts as well as cells grown with a higher concentration of PLO (Fig. 4). Contrastingly, *RFX3* expression was higher in 0.01 % PLO cultures on both days. Expression of other genes such as *NKX6.1, NKX2.2, NEUROD1, PAX4, SOX17, PAX6, GCG* and *PPY* varied with culture condition and day of culture although their expression was significantly higher than their non-PLO counterparts. In general, the combined effect of EGF and PLO caused an increase in expression of the early gene markers such as *PAX4*, *PAX6* and *SOX17*. By the 7th day, the expression of these genes fell whereas those of later genes such as *NGN3* and *NEUROD1* increased although *NGN3* expression was not statistically significant.

Under the microscope it was observed that the lower concentration of PLO was less cytotoxic (Additional file 7).

## Discussion

The human amnion is a foetal membrane that surrounds the placenta and is derived from the epiblast as early as 8 days after fertilization [21]. There are 255 births worldwide every minute [22]. More than half of the placentas obtained post-partum are discarded. Since the placenta and its surrounding membranes act as a maternal-foetal interface, it is quite likely that multipotent progenitor cells can be obtained from them. Several studies have in fact, shown the extraordinary differentiation potential of placenta derived multi progenitor cells and human amnion epithelial cells into cells of all three dermal lineages including the endocrine pancreas, neuronal cells etc. [8, 9, 14, 23, 24]. The present study has added to the knowledge obtained from such studies.

The complex development of the pancreas involves a fine play between several transcription factors, chief amongst which is the transcription factor Pdx1 [10–12]. In the present study, endogenous h*PDX1* expression was activated. This caused cells to stop proliferating and to undergo differentiation instead.

Once *PDX1* expression was activated, many other downstream genes involved in the pancreatic differentiation process were also activated, chief amongst which was the dramatic expression of *RFX6*. Although Rfx6 has been suggested to be an Ngn3-dependent transcription factor [25], we observed a significant, albeit low level of *NGN3* expression only at the 7 day time point by which time *RFX6* expression had decreased 3-fold. A significant increase in expression of other genes downstream of *NGN3* such as *NEUROD1* and *PAX4* were also observed at this point, although the expression of these genes was very low in terms of Cq.

General endoderm marker, *SOX17*, and anterior endoderm marker, *FOXA2*, expression were increased. It was observed that as the expression of these two genes increased over the 7 day period, there was a corresponding loss of *RFX6* expression. This is consistent with Pearl et. al’s observation in the *Xenopus* system where *RFX6* was expressed in two waves, the first of which occurs in the anterior endoderm, up stream of *NGN3* expression[26]. However they also positioned Rfx6 up stream of Pdx1 since they observed lower gene expression of *PDX1* in *RFX6* knockouts. Our observation differed in that we did not see a loss of *PDX1* expression as *RFX6* expression reduced. In fact, *PDX1* expression continued to increase over the 7 days in cultures containing EGF and 0.01 % PLO. Corroborating evidence is provided by Suzuki et al. who observed significant loss of Rfx6 expression upon Pdx1 silencing in mouse intestinal epithelial cells but did not see any significant effect of Rfx6 silencing on Pdx1 expression [27]. This suggests that some of our cells were still in the early stages of pancreatic development similar to Soyer J et al.’s observation that *Rfx6* is initially expressed in the Pdx1-positive gut endoderm cells in mice and zebrafish [25].

Upon the extended culture time period, the expression of the multipotent pancreatic duct-like cell markers of *FOXA2* and *CFTR* increased. While there was no significant expression of endocrine cell markers such as *MAFA* and *INS*, there was a greater than 10-fold increase in expression of the alpha cell marker, *GCG* and PP cell marker, *PPY.* EGF alone could sustain this expression over the entire duration of culture.

Since hAECs have a high endogenous *CK19* and *SOX9* expression (Fig. 1), the fold-change in mRNA expression of these two genes upon adenoviral transduction even with culture on PLO, was not significant. However, we hypothesize that the high endogenous expression of *CK19* and *SOX9*, coupled with the induced expression of *PDX1* and other pancreatic marker genes, lead the cells down the pancreatic lineage. Indeed, since the cells were positive for both *SOX9* and *PDX1*, markers of the posterior foregut, we hypothesize that some of the cells are similar to E10.5-11.5 mouse embryonic pancreatic epithelium [12] or primary pancreatic progenitor cells [28]. Interestingly, Wescott et al. observed that Pdx1^hi^/insulin-negative cells may represent branching epithelia [12] and Cardinale V et al. identified multipotent stem/progenitors in high numbers in the hepato-pancreatic common duct [29]. The cells which they identified were positive for endodermal transcription factors viz. *SOX9, SOX17, FOXA2, PDX1, NGN3*; stem/progenitor surface markers viz. *EpCAM, NCAM, CD133, CXCR4*; and sometimes weakly, adult liver, bile duct, and pancreatic genes viz. albumin, *CFTR* and insulin, respectively. Since we were able to observe expression of some these markers in our cells as well, it provides further evidence to the possibility of our cells representing a mixed population of cells along varying degrees of pancreatic differentiation some of which are similar to the multipotent progenitors of the pancreatic duct.

Although earlier studies have indicated that a lack of EGF in the culture environment drives the differentiation process [16], there are some others which have suggested a role for EGF and its family members in establishing islet architecture [10]. In the present study we found that EGF was required to sustain and in some cases even increase the expression of pancreatic marker genes. While a lack of EGF reduced the stemness of the differentiating hAECs, it also caused a significant loss of later stage pancreatic markers including the alpha cell marker, *GCG*.

Human and non-human primate amniotic fluid-derived stem cells (AFSCs) have previously been induced to express *PDX1* when cultured on PLO-coated plates. This was shown to initiate pancreatic differentiation [5, 6]. In this study, it was observed that while PLO itself did not initiate pancreatic differentiation, it greatly potentiated pancreatic differentiation upon m*Pdx1* transduction. What was interesting to observe was that in conditions where a lack of EGF resulted in a higher expression of a particular gene marker, a concentration of 0.01 % PLO provided similar results. When EGF or a lower concentration of PLO was introduced into the culture environment, the gene expression levels fell. It seems therefore, that a delicate balance between EGF and PLO needs to be achieved in order to drive differentiation as opposed to the maintenance of cell status.

With the incidence of diabetes on the rise and current therapy still being inadequate in preventing disease progression, the bioartificial pancreas could well be the future of diabetes therapy with enormous potential for a cure. The in vivo differentiation potential of the heterogenous population of pancreatic progenitors derived from multipotent hAECs in this present study is warranted as it could suggest their future use for cellular therapy for insulin-dependent diabetes.

## Conclusion

Since the presence of true pancreatic stem cells is still a matter of debate, the plasticity of other, easily available multipotent cells could be important for a cell-therapy based approach to treat insulin-dependent diabetes. We propose human amnion epithelial cells that have been engineered to over express *PDX1*, as potential sources of pancreatic multipotent progenitors and thus as precursors for endocrine pancreas development. This process can be further potentiated with a mix of epidermal growth factor and poly-L-ornithine in the culture environment. This has important implications in the field of diabetes therapy.

## Methods

### Culture of cells

HyClone™ Dulbecco’s Low Glucose Modified Eagles Medium (DMEM-LG, catalog # SH30021) and HyClone™ Non-Essential Amino Acids (NEAA, catalog # SH30238) were obtained from GE Healthcare Life Sciences. 100X Insulin, Transferrin, Selenite Liquid Media Supplement (ITS, catalog # I3146) was from Sigma-Aldrich, USA. Foetal Bovine Serum (FBS, catalog # 10082), Epidermal Growth Factor (EGF, Recombinant Human Protein; catalog # PHG0311) and Penicillin-Streptomycin (catalog # 15070) were obtained from Gibco™.

Uncultured (p0) human amnion epithelial cells (hAECs) were kindly provided by Dr. Sean Murphy (WFIRM, USA). Experimental cells were cultured up to passage 2 (p2) in complete medium (DMEM-LG supplemented with 10%FBS, 1 % NEAA, 1 % ITS, 10 ng/mL EGF and 1 % penicillin-streptomycin), under standard cell culture conditions (5 % CO_2_/37 °C). Control cells were cultured without EGF at passage 2.

Human pancreases were procured from deceased donors under an MOU with the Carolina Donor Services (CDS) for the supply of organs from individuals who had signed up to donate their organs for research. The CDS is the federally designated organ procurement organization, including hospitals and transplant centers that perform heart, lung, liver, kidney, pancreas and intestine transplantation. Donated organs are processed after obtaining authorization from the donor or their family.

Crude preparations of adult human islets from donor pancreases were made by a modified method of Ricordi C et al. [30]. Isolated tissue pellet was immediately frozen without any preservation media at −80 °C until use for RNA extraction. The research was approved by the Institutional Review Board committee of the Wake Forest School of Medicine (#IRB00028826).

### Adenoviral transduction and study on the effect of EGF

Adenovirus expressing mouse Pdx1 (mPdx1) was a gift from Drs. Christopher Newgard and Sarah Ferber at Duke University.

To confirm adenoviral transduction by fluorescence microscopy, p0 hAECs were seeded at a density of 5,000 cells/cm^2^ in chamber slides (1.7 cm^2^ per chamber) and cultured under standard conditions overnight. The next morning, the cultures were washed twice with DPBS or plain DMEM-LG and the appropriate viral titre diluted in 500 μL plain DMEM-LG was added. Four titres were used—10, 50, 100 and 200 MOI. After 6 h under standard culture conditions, an equal volume of complete medium was added to the cultures and cells were cultured thus overnight. The next morning, virus containing media was removed and cells were washed twice with DPBS or plain DMEM-LG. Two millilitre complete medium was added to each chamber and cells were cultured under standard conditions. The cells were fixed and immunostained 24 h and 48 h after removal of virus-containing media. Primary antibody used for mPdx1 immunofluorescence staining was a monoclonal anti-mouse Pdx1 antibody (R&D systems, USA; catalog # MAB2419). Secondary antibody was horse anti-mouse IgG-Texas red conjugated antibody (H + L; Vector Labs, USA; catalog # TI-2000). All nuclei were counter stained with DAPI (Vector Labs, USA; catalog # H-1200). Number of Texas red and DAPI stained nuclei were counted manually in two different fields. Efficiency of transduction was then estimated as:

$$ \%\  transduction = \left( Number\  of\  Texas\  red\  stained\  nuclei/ Number\  of\  DAPI\  stained\  nuclei\right) \times 100 $$

For RNA isolation and qPCR experiments, p2 hAECs that had achieved confluence were trypsinized, counted and plated in 6-well plates at a seeding density of 10,000 cells/cm^2^. Adenoviral transduction with 50 MOI of m*Pdx1*-adenovirus was carried out as described above. One set of cultures lacked EGF in the complete medium. Cells were cultured for a further 2 days or 7 days in standard culture medium before RNA was extracted from them. Cells were also observed microscopically daily in order to determine if there were any accompanying morphological changes. At the time of cell culture, cultures were performed in biological triplicates.

### Adenoviral transduction and study on the combined effect of EGF and PLO

One set of plates were either coated with 0.01 % or 0.001 % Poly-L-Ornithine (PLO; Sigma-Aldrich, catalog # P2533) according to the CSH protocols [31]. Cells were then cultured and transduced with m*Pdx1*-adenovirus as described above. Cells were cultured for a further 2 days or 7 days in standard culture medium containing 10 ng/mL EGF. Cells were also observed microscopically daily in order to determine if there were any accompanying morphological changes. At the time of cell culture, cultures were performed in biological triplicates.

### RNA extraction

At the 2 day or 7 day time point, RNA was isolated from the experimental and control cells using the 5 PRIME manual PerfectPure^TM^ RNA Cultured Cell Kit (5 PRIME, Inc., USA; catalog # 2302340). Since it was found that RNA yield from cells in one well of the 6-well plate was low, RNA from 3 wells were pooled together to represent one sample.

RNA was also isolated from p2 hAECs that had been grown in the absence of adenovirus, EGF and PLO. RNA was isolated from frozen adult human islets when required.

Total RNA content was estimated using a Nanodrop 2000c (Thermo Scientific, USA).

### cDNA synthesis

500 ng of total RNA was used per 20 μL reaction, to synthesise cDNA using the High-Capacity cDNA Reverse Transcription Kit (Applied Biosystems, USA; catalog # 4368813). PCR conditions were as provided by the manufacturer’s protocol i.e. 25 °C for 10 min, 37 °C for 2 h and 85 °C for 5 min.

### Primers for qPCR

Stem cell marker: *OCT4, NANOG;* Early/Definitive endoderm markers: *SOX17, PAX6*; Anterior endoderm marker: *FOXA2*; Posterior foregut markers: *PDX1*, *SOX9*; Endocrine progenitor markers: *NGN3, NEUROD1, NKX2.2, NKX6.1, RFX6, RFX3, PAX4*; Endocrine pancreas markers: *MAFA, INS, GCG, PPY*; Pancreatic duct markers: *FOXA2, CFTR, CK19;* Housekeeping gene marker: *GAPDH.*

The sequence of the primers that were obtained from Integrated DNA technologies (IDT, USA) and purified by standard desalting are given in Table 1. The specificity of the primers for the particular target gene was evaluated *in silico* using the Primer BLAST tool [32].

**Table 1:** Primers for genes whose expression was evaluated by the SYBR® Green method

Gene (GenBank ID)	F primer sequence (5′ to 3′)	R primer sequence (5′ to 3′)	Expected product size
*RFX6* (306518575)	TCTCTTTGACCAGCATGTCG	CTGTGCTGCCTGAAATGGTA	104 bp spanning region within exon 12
*CFTR* (306514)	CTATGACCCGGATAACAAGGAGG	CAAAAATGGCTGGGTGTAGGA	107 bp spanning region within exon 4 of all transcript variants
*CK19* (239735540)	TTTGAGACGGAACAGGCTCT	CTCGGCCATGACCTCATATT	279 bp spanning region within exon 3
*SOX9* (758102)	AAGCTCTGGAGACTTCTGAA	TAACGGGGCTCACGAGCGGC	271 bp spanning exons 1 and 2 in the CDS
*RFX3* (Harvard PrimerBank ID 19743882c2)	CCAGGTGACTACCGTGGTCT	GCTGCTGATGAGTTGTCCTCC	88 bp spanning region within exon 1

The table lists the genes (and their corresponding GenBank IDs) whose expression was evaluated by the SYBR® Green method. The corresponding forward (F) and reverse (R) primer sequences and the region it spans in the target gene are also mentioned

### Quantitative real-time PCR (qPCR)

cDNA equivalent to 12.5 ng of initial RNA was used per 20 μL reaction for qPCR analysis. *RFX6*, *RFX3*, *CFTR*, *CK19* and *SOX9* expression were all estimated using the SYBR® Green method. 250 nanomolar of each primer (forward or reverse) was used per reaction. In the case of hydrolysis probes (Table 2), 1 μL of the appropriate 20X TaqMan® Hydrolysis probe mix was used per reaction. Ten microliters of the 2X SYBR® Green PCR Master Mix (Applied Biosystems, USA; catalog # 4309159) or 2X TaqMan® Gene Expression Master Mix (Applied Biosystems, USA; catalog # 4369016) was added to the appropriate reaction mixes and made up to 20 μL with nuclease-free water. Reactions were set up in MicroAmp® Optical 96-Well Reaction Plates (Applied Biosystems, USA; catalog # 4306737) and plates were sealed with MicroAmp® Optical Adhesive Film (Applied Biosystems, USA; catalog # 4360954). qPCR was performed on an Applied Biosystems® 7500 Real-time PCR system. Default PCR conditions were used (50 °C for 2 min, 95 °C for 10 min, 40 cycles of 95 °C for 15 s and 60 °C for 1 min. Dissociation: 95 °C for 15 s, 60 °C for 20 s, 95 °C for 15 s and 60 °C for 15 s). All qPCR reactions were carried out as technical replicates. Relative gene expression was estimated manually using the ddCt method [33, 34]. h*GAPDH* was used as the reference gene for all experiments. Unmanipulated p2 hAEC (untransduced; grown in the absence of EGF and PLO) gene expression was the quantification calibrator (baseline gene expression) for experiments in which the effect of m*Pdx1* transduction was studied. For all other experiments, m*Pdx1*-transduced hAECs grown in the presence of EGF was used as the quantification calibrator. All results are represented according to the MIQE guidelines [35].

**Table 2:** Primers for genes whose expression was evaluated by the TaqMan® method

Serial no.	Gene symbol	Catalog number
1	*OCT4*	Hs03005111_g1
2	*NANOG*	Hs02387400_g1
3	*SOX17*	Hs00751752_s1
4	*PAX6*	Hs01088112_m1
5	*FOXA2*	Hs00232764_m1
6	*PDX1*	Hs00426216_m1
7	*NGN3*	Hs00360700_g1
8	*NEUROD1*	Hs00159598_m1
9	*NKX2.2*	Hs00159616_m1
10	*NKX6.1*	Hs00232355_m1
11	*PAX4*	Hs00173014_m1
12	*MAFA*	Hs01651425_s1
13	*INS*	Hs00355773_m1
14	*GCG*	Hs00174967_m1
15	*PPY*	Hs00237001_m1
16	*GAPDH*	Hs99999905_m1

The table lists the genes which were evaluated by the TaqMan® method. The corresponding primers were validated hydrolysis probes from Applied Biosystems, USA

For the present study, gene expression in terms of quantification cycle (Cq) was classified as high (Cq <25), medium (Cq between 25 and 30), low (Cq between 30 and 35) and no expression (Cq ≥ 35). For each culture condition, only those genes that had a Cq <35 are represented graphically.

For individual gene expression under each culture condition, the statistical significance was estimated by a two tailed *t*-test (assuming equal variances) in Microsoft Excel. The 2^-Cq values for each replicate were used for the calculations. Alpha was set at 0.05.

## Ethics approval and consent to participate

Human pancreases were procured from deceased donors under an MOU with the Carolina Donor Services (CDS) for the supply of organs from individuals who had signed up to donate their organs for research. The CDS is the federally designated organ procurement organization, including hospitals and transplant centers that perform heart, lung, liver, kidney, pancreas and intestine transplantation. Donated organs are processed after obtaining authorization from the donor or their family.

The use of human amnion epithelial cells was approved by the Institutional Review Board committee of the Wake Forest School of Medicine (#IRB00002852). The use of human islets was approved by the Institutional Review Board committee of the Wake Forest School of Medicine (#IRB00028826).

## Consent for publication

Not applicable

## Availability of data and materials

The data sets supporting the results of this article are included as additional files.

## Additional files

Additional file 1: Figure S1. Comparison of transduction efficiency of various adenoviral titres. (a) hAECs were transduced with adenovirus harbouring an m*Pdx1* vector at various MOIs (multiplicity of infection). Cells were stained with an mPdx1-specific antibody (Texas-Red conjugate) at 24 h and 48 h post infection to determine the transduction efficiency. Nuclei were counter stained with DAPI (blue). (b) Calculation of transduction efficiency from two different microscopic fields of cells 24 h after transduction with 50 MOI of the m*Pdx1*-harbouring adenovirus. Cells were viewed using the 10X objective of an Olympus inverted fluorescence microscope. Purple nuclei are those that are stained by both DAPI and Texas-Red conjugated secondary antibody. (ZIP 1201 kb) Additional file 2: Effect of m*Pdx1* transduction on p2 hAECs. The qPCR data obtained upon transducing p2 hAECs with 50MOI of the m*Pdx1* adenovirus is given. The statistical calculations are also included. (XLSX 67 kb) Additional file 3: Figure S2. Microscopic comparison of *EGFP* and m*Pdx1* transduced cells. Cells that were transduced with either *EGFP* or m*Pdx1* were observed 2, 4 and 7 days post-transduction. It was observed that while EGFP-expressing cells continued to proliferate over the 7 day period, proliferation ceased in cells transduced with m*Pdx1*. Cells transduced with m*Pdx1* thus seemed to stop the process of proliferation and continued the process of differentiation instead. Cells were viewed using the 10X objective of an Olympus inverted fluorescence microscope. (PNG 1561 kb) Additional file 4: Figure S4. Western blot analysis. In order to prove protein expression of some of the gene expression observed by qPCR, Western blot analysis was carried out with anti-RFX6 and anti-RFX3 antibodies. (a) Comparison of the nuclear and cytoplasmic extract of adult human islets and untransduced hAECs. The islet nuclear extract (INE) and to some extent the cytoplasmic (ICE) extract stained positive for RFX6. hAEC nuclear (AECNE) and cytoplasmic (AECCE) extract were negative for RFX6 staining. This is in corroboration with qPCR analysis from Fig. 1. (b) Cell lysate from day 2 mPdx1 transduced cells grown in the presence of EGF were run on a 10 % SDS-PAGE and then immunoblotted with an anti-RFX3 or anti-RFX6 antibody. RFX3 signal was stronger than RFX6 corresponding to the higher expression of the former seen even in qPCR experiments. Anti-RFX6 antibody (Rabbit polyclonal, Sigma, catalog # SAB1402062) and anti-RFX3 antibody (Mouse polyclonal, Sigma, catalog # SAB1400241) were used at 1:1000 dilutions. Corresponding secondary antibodies used were anti- rabbit IgG-HRP conjugate (Cell signaling, catalog # 7074 s, 1:1000 dilution) and anti-mouse IgG-HRP conjugate (Cell signaling, catalog # 7076, 1:1000 dilution). The images were developed using the SuperSignal™ West Femto Maximum Sensitivity Substrate (Thermo Scientific^TM^, catalog # 34094). (PNG 169 kb) Additional file 5: Effect of EGF on m*Pdx1* transduced hAECs. The qPCR data obtained upon transducing p2 hAECs with 50MOI of the m*Pdx1* adenovirus is given. One set of cells were grown in the absence of EGF. The statistical calculations are also included. (XLSX 66 kb) Additional file 6: Combined effect of EGF and PLO on m*Pdx1* transduced hAECs. The qPCR data obtained upon transducing p2 hAECs with 50MOI of the m*Pdx1* adenovirus is given. One set of cells were grown in the presence of 0.01 % PLO while another was grown in the presence of 0.001 % PLO. All cells were grown in the presence of 10 ng/mL EGF. The statistical calculations are also included. (XLSX 108 kb) Additional file 7: Figure S3. Microscopic observation of cells. Cells that were transduced with m*Pdx1* and cultured under various culture conditions were observed at the time of transduction (0th hour) and 2 days and 7 days post-transduction. It was observed that although all cells were equally healthy at the 0th hour, the various culture conditions caused changes in their health, although not morphology, from the 2nd day post-transduction. In general, cells started senescing by the 7th day with 0.01 % PLO being the most cytotoxic of all culture conditions. Cells were viewed using the 10X objective of an Olympus inverted fluorescence microscope. (PNG 763 kb)

## Abbreviations

EGF: epidermal growth factor
hAECs: human amnion epithelial cells
Pdx1: pancreatic and duodenal homeobox-1
PLO: poly-L-ornithine
