# Supplementary figures and images for: The combined effect of PDX1, epidermal growth factor and poly-L-ornithine on human amnion epithelial cells’ differentiation
Source: BMC Dev Biol. 2016 Apr 12;16:8. doi: 10.1186/s12861-016-0108-y (PMC4828805; doi:10.1186/s12861-016-0108-y)

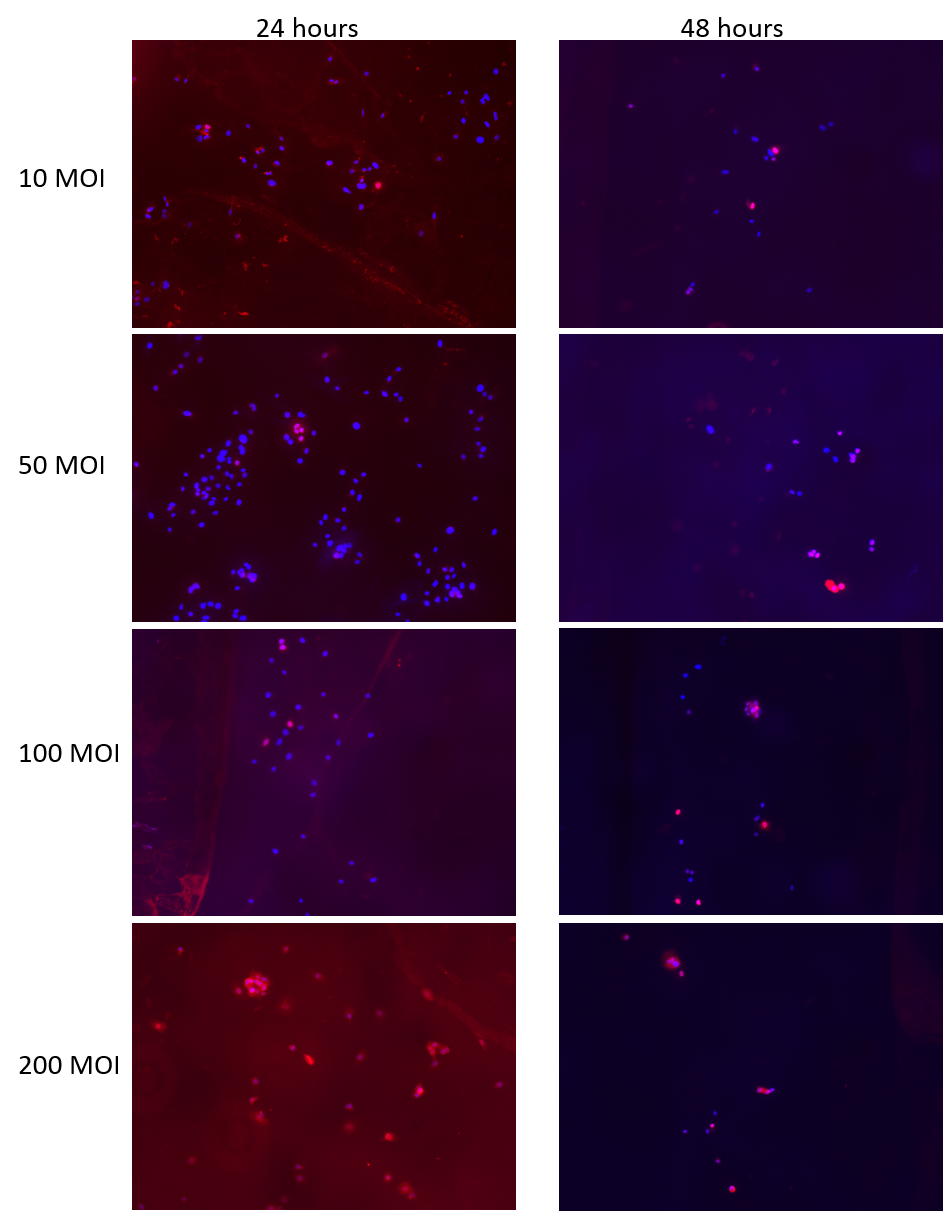

Supplement: Additional file 1: Figure S1. — Comparison of transduction efficiency of various adenoviral titres. (a) hAECs were transduced with adenovirus harbouring an mPdx1 vector at various MOIs (multiplicity of infection). Cells were stained with an mPdx1-specific antibody (Texas-Red conjugate) at 24 h and 48 h post infection to determine the transduction efficiency. Nuclei were counter stained with DAPI (blue). (b) Calculation of transduction efficiency from two different microscopic fields of cells 24 h after transduction with 50 MOI of the mPdx1-harbouring adenovirus. Cells were viewed using the 10X objective of an Olympus inverted fluorescence microscope. Purple nuclei are those that are stained by both DAPI and Texas-Red conjugated secondary antibody. (ZIP 1201 kb) [file 12861_2016_108_MOESM1_ESM.zip › Supplmentary Picture 1a,b/Supplementary Picture 1.png]

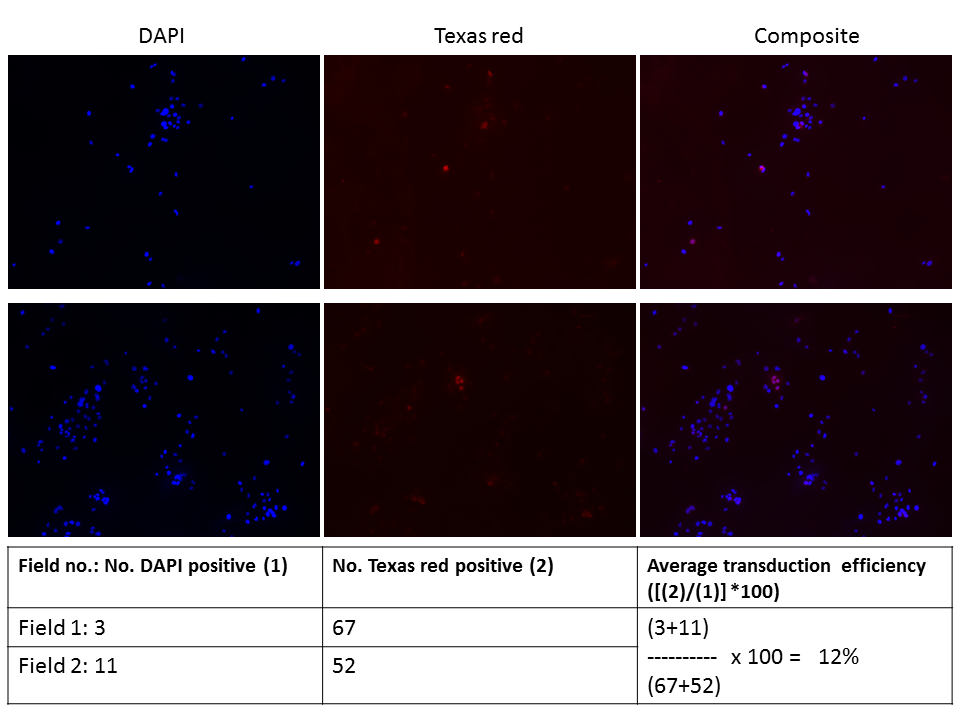

Supplement: Additional file 1: Figure S1. — Comparison of transduction efficiency of various adenoviral titres. (a) hAECs were transduced with adenovirus harbouring an mPdx1 vector at various MOIs (multiplicity of infection). Cells were stained with an mPdx1-specific antibody (Texas-Red conjugate) at 24 h and 48 h post infection to determine the transduction efficiency. Nuclei were counter stained with DAPI (blue). (b) Calculation of transduction efficiency from two different microscopic fields of cells 24 h after transduction with 50 MOI of the mPdx1-harbouring adenovirus. Cells were viewed using the 10X objective of an Olympus inverted fluorescence microscope. Purple nuclei are those that are stained by both DAPI and Texas-Red conjugated secondary antibody. (ZIP 1201 kb) [file 12861_2016_108_MOESM1_ESM.zip › Supplmentary Picture 1a,b/Supplementary Picture 1b.png]

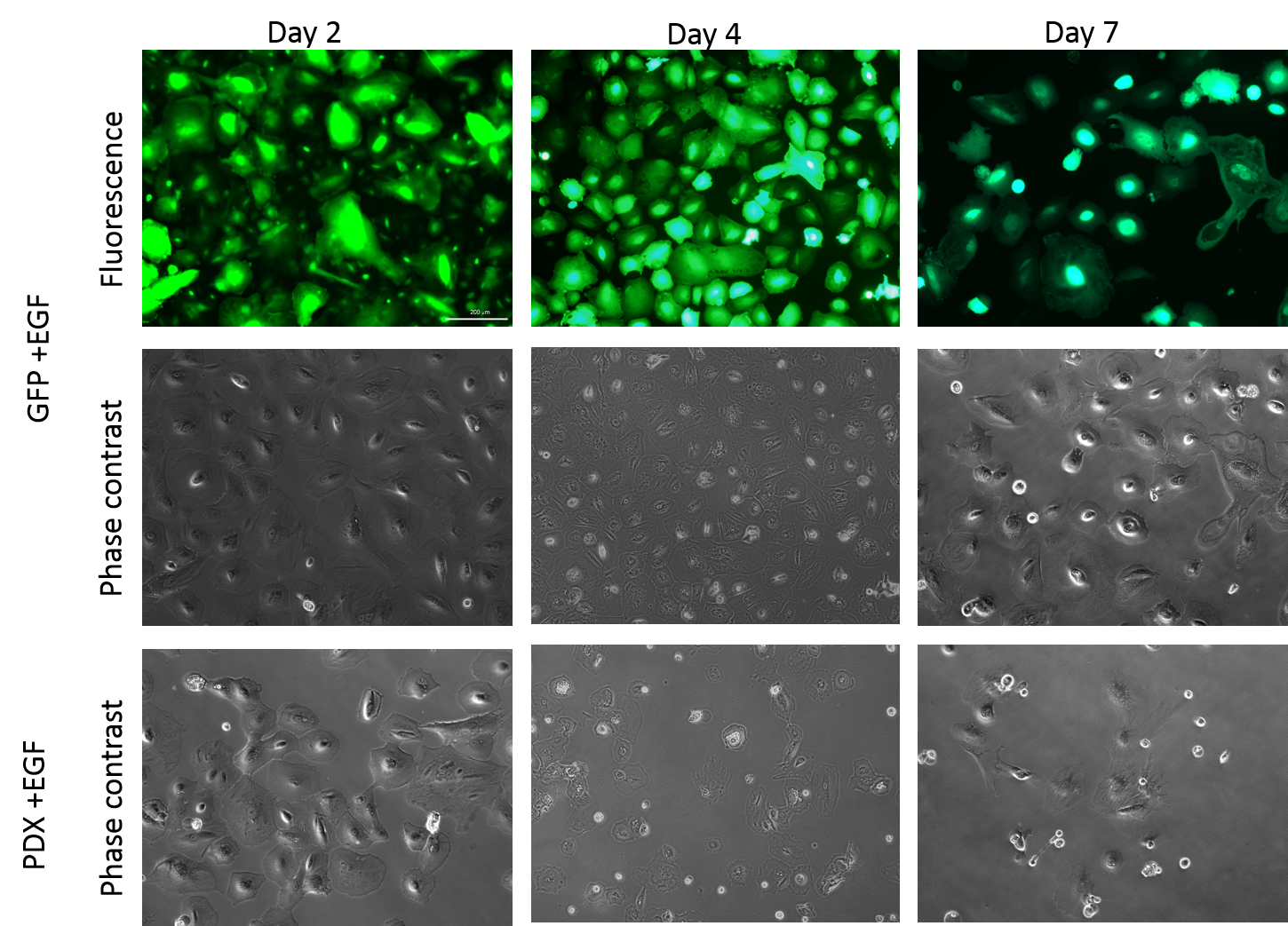

Supplement: Additional file 3: Figure S2. — Microscopic comparison of EGFP and mPdx1 transduced cells. Cells that were transduced with either EGFP or mPdx1 were observed 2, 4 and 7 days post-transduction. It was observed that while EGFP-expressing cells continued to proliferate over the 7 day period, proliferation ceased in cells transduced with mPdx1. Cells transduced with mPdx1 thus seemed to stop the process of proliferation and continued the process of differentiation instead. Cells were viewed using the 10X objective of an Olympus inverted fluorescence microscope. (PNG 1561 kb) [file 12861_2016_108_MOESM3_ESM.png]

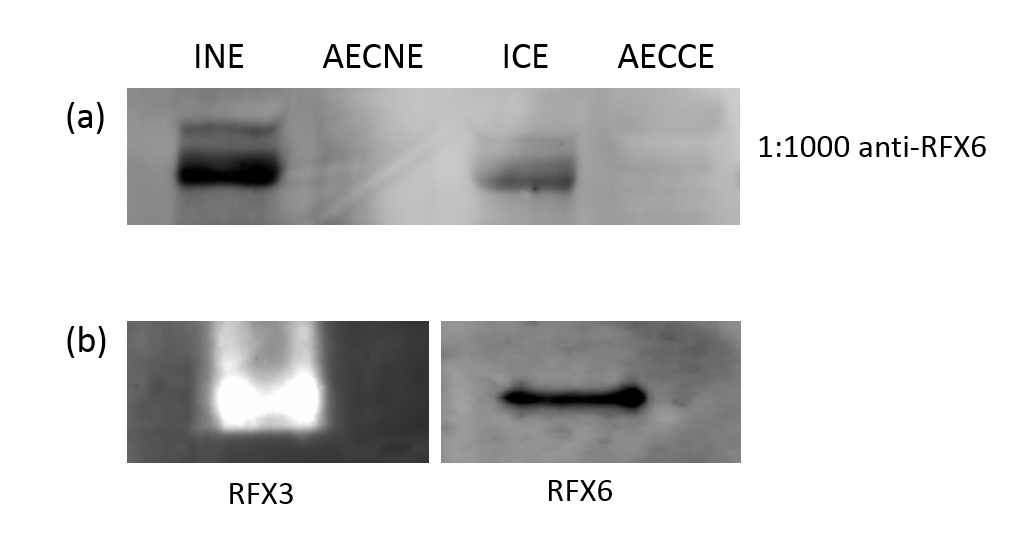

Supplement: Additional file 4: Figure S4. — Western blot analysis. In order to prove protein expression of some of the gene expression observed by qPCR, Western blot analysis was carried out with anti-RFX6 and anti-RFX3 antibodies. (a) Comparison of the nuclear and cytoplasmic extract of adult human islets and untransduced hAECs. The islet nuclear extract (INE) and to some extent the cytoplasmic (ICE) extract stained positive for RFX6. hAEC nuclear (AECNE) and cytoplasmic (AECCE) extract were negative for RFX6 staining. This is in corroboration with qPCR analysis from Fig. 1. (b) Cell lysate from day 2 mPdx1 transduced cells grown in the presence of EGF were run on a 10 % SDS-PAGE and then immunoblotted with an anti-RFX3 or anti-RFX6 antibody. RFX3 signal was stronger than RFX6 corresponding to the higher expression of the former seen even in qPCR experiments. Anti-RFX6 antibody (Rabbit polyclonal, Sigma, catalog # SAB1402062) and anti-RFX3 antibody (Mouse polyclonal, Sigma, catalog # SAB1400241) were used at 1:1000 dilutions. Corresponding secondary antibodies used were anti- rabbit IgG-HRP conjugate (Cell signaling, catalog # 7074 s, 1:1000 dilution) and anti-mouse IgG-HRP conjugate (Cell signaling, catalog # 7076, 1:1000 dilution). The images were developed using the SuperSignal™ West Femto Maximum Sensitivity Substrate (Thermo ScientificTM, catalog # 34094). (PNG 169 kb) [file 12861_2016_108_MOESM4_ESM.png]

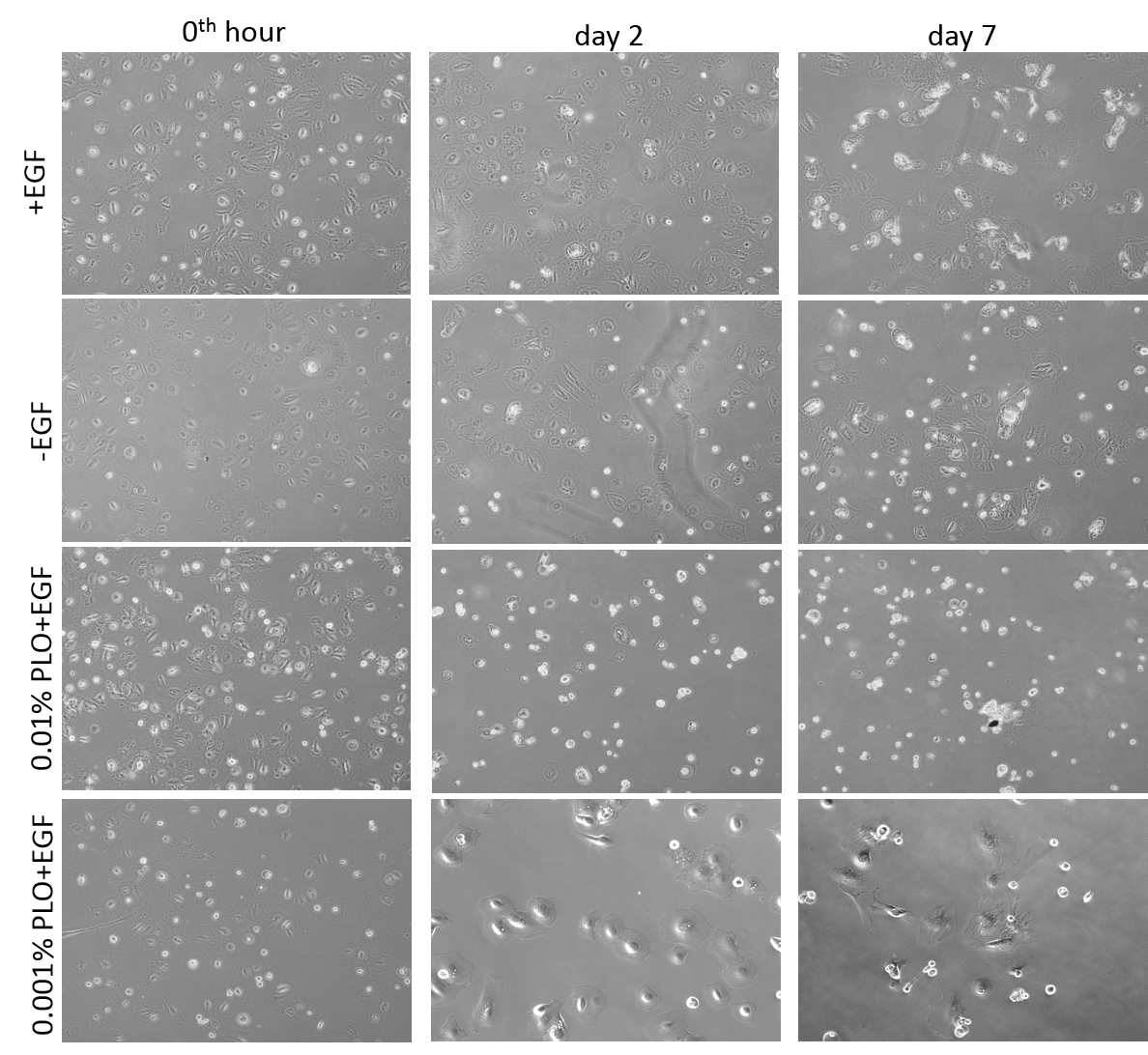

Supplement: Additional file 7: Figure S3. — Microscopic observation of cells. Cells that were transduced with mPdx1 and cultured under various culture conditions were observed at the time of transduction (0th hour) and 2 days and 7 days post-transduction. It was observed that although all cells were equally healthy at the 0th hour, the various culture conditions caused changes in their health, although not morphology, from the 2nd day post-transduction. In general, cells started senescing by the 7th day with 0.01 % PLO being the most cytotoxic of all culture conditions. Cells were viewed using the 10X objective of an Olympus inverted fluorescence microscope. (PNG 763 kb) [file 12861_2016_108_MOESM7_ESM.png]
